# Interpreting Venous and Arterial Ulcer Images Through the Grad-CAM Lens: Insights and Implications in CNN-Based Wound Image Classification

**DOI:** 10.3390/diagnostics15172184

**Published:** 2025-08-28

**Authors:** Hannah Neuwieser, Naga Venkata Sai Jitin Jami, Robert Johannes Meier, Gregor Liebsch, Oliver Felthaus, Silvan Klein, Stephan Schreml, Mark Berneburg, Lukas Prantl, Heike Leutheuser, Sally Kempa

**Affiliations:** 1 Department of Plastic, Hand, and Reconstructive Surgery, University Hospital Regensburg, 93053 Regensburg, Germany; 2 Ambient Assisted Living & Medical Assistance Systems, Department of Computer Science, University of Bayreuth, 95447 Bayreuth, Germany; 3 Machine Learning and Data Analytics Lab (MaD Lab), Department Artificial Intelligence in Biomedical Engineering (AIBE), Friedrich-Alexander-Universität Erlangen-Nürnberg (FAU), 91052 Erlangen, Germany; 4 Imaging Solutions, PreSens Precision Sensing GmbH, 93053 Regensburg, Germany; 5 Department of Dermatology, University Hospital Regensburg, 93053 Regensburg, Germany

**Keywords:** deep learning, artificial intelligence, wound classification, arterial ulcers, venous ulcers, convolutional neural networks

## Abstract

**Background/Objectives**: Chronic wounds of the lower extremities, particularly arterial and venous ulcers, represent a significant and costly challenge in medical care. To assist in differential diagnosis, we aim to evaluate various advanced deep-learning models for classifying arterial and venous ulcers and visualize their decision-making processes. **Methods**: A retrospective dataset of 607 images (198 arterial and 409 venous ulcers) was used to train five convolutional neural networks: ResNet50, ResNeXt50, ConvNeXt, EfficientNetB2, and EfficientNetV2. Model performance was assessed using accuracy, precision, recall, F1-score, and ROC-AUC. Grad-CAM was applied to visualize image regions contributing to classification decisions. **Results**: The models demonstrated high classification performance, with accuracy ranging from 72% (ConvNeXt) to 98% (ResNeXt50). Precision and recall values indicated strong discrimination between arterial and venous ulcers, with EfficientNetV2 achieving the highest precision. **Conclusions**: AI-assisted classification of venous and arterial ulcers offers a valuable method for enhancing diagnostic efficiency.

## 1. Introduction

Chronic wounds, particularly venous and arterial ulcers of the lower extremities, represent a global problem with a prevalence estimated at between 0.1% and 2% in the general population [1,2]. As society continues to age and the obesity rate increases, vascular ulcers are also anticipated to rise because of obesity-associated comorbidities. Timely diagnosis of this condition is crucial for selecting the appropriate therapy and preventing further progression of the wound, as well as avoiding amputation of the limb, which is a not uncommon complication of this wound type [3]. In general, arterial and venous ulcers have numerous classic features that help with differential diagnosis [4]. The cause of an arterial ulcer is peripheral arterial disease (PAD), i.e., the arteries supplying the affected extremity are either stenosed or completely occluded. Due to this reduced blood flow, minor traumas heal poorly and thus lead to the classic clinical picture of a well-defined ulcer with deep (“punched-out”) wound edges. Due to tissue hypoxia, the wounds tend to be dry and often show black necrotic tissue or exposed tendons, suggesting a non-venous condition. In contrast, venous ulcers tend to be more irregular in shape and have a rather moist wound environment. While venous ulcers often occur above or behind the medial malleolus, arterial ulcers tend to occur on the edge of the tibia, above the lateral malleolus, or on the feet. The surrounding skin also differs (Table 1).

Clinical signs, patient history, and vascular imaging are recommended to determine the etiology of the ulcer. The primary tests for diagnosing arterial and venous ulcers are the ankle-brachial pressure index (ABPI), duplex ultrasonography, and color flow Doppler imaging (CFDI) [5,6]. Further examinations, such as venography, angiography, microbiological and/or histological examination of a biopsy, may be necessary in some cases to clarify the etiology of the ulcer. While this offers essential insights into the etiology of the wound, it is also time-consuming, subjective, and requires specialized training, delaying proper therapy beyond local wound care. Furthermore, not all care facilities have access to comprehensive diagnostic tools like duplex ultrasound. In such settings, AI-based wound classification could offer a quick and reliable way to determine the cause, supported by clear visual explanations such as Grad-CAM. This could, for example, enable early identification of patients likely to have venous ulcers, allowing them to benefit immediately from compression therapy. Therefore, AI could serve as a practical decision-support tool that helps clinicians in the diagnostic process and may speed up the initiation of appropriate treatment.

Artificial intelligence (AI), Machine learning (ML), and Deep learning (DL) have revolutionized medical image analysis. For example, in dermatology, the diagnosis and risk assessment of skin changes in preventive care are automated and specific [7]. In radiological imaging, AI can enhance image quality [8] and help detect and classify anomalies [9,10]. DL, a subfield of ML, employs deep neural networks to identify patterns. Convolutional neural networks, a prominent area in DL, effectively process two-dimensional input data.

Recent studies increasingly move from single-word outputs toward paragraph-level report generation, enabled by vision-language models that couple strong visual backbones with text decoders. These systems highlight clinically aligned evaluation and factuality and outline clear pathways from robust classifiers to structured findings and auto-drafted reports [11,12,13]. In this context, our focus on reliable ulcer classification serves as a practical building block for future report-level outputs.

Deep convolutional neural networks (DCNNs) are a variant of convolutional neural networks (CNNs) that can recognize relevant image details and more complicated patterns in the analysis of images. In wound assessment, there are various approaches for identifying wound boundaries (localization or segmentation) and classifying different wound types [14,15] and tissue types (e.g., granulation, slough, and eschar) [14] using DL.

So far, however, the classification has been broader between different wound types, such as surgical wounds, venous leg ulcers, and pressure ulcers [14]. A decision support system for a detailed distinction between venous and arterial ulcers could contribute to a faster diagnosis and, thus, earlier targeted therapy. Additionally, employing explainable AI (XAI) modeling can facilitate its integration and acceptance within healthcare systems [16,17].

Our study aims to apply and evaluate various state-of-the-art deep learning models for the image-based classification of vascular ulcers, specifically distinguishing between venous and arterial ulcers. Beyond classification performance, our clinical focus lies in visualizing and interpreting the decision-making process of the model using explainable AI (XAI) techniques such as Gradient-weighted Class Activation Mapping (Grad-CAM), to ensure transparency and facilitate clinical trust and integration.

## 2. Materials and Methods

### 2.1. Patients

We utilized the hospital database from University Hospital Regensburg (Germany) to retrospectively filter all patients by the relevant ICD codes (International Statistical Classification of Diseases and Related Health Problems) for chronic ulcers of the lower extremities. Next, we manually verified their diagnoses in the patient charts based on vascular assessments (e.g., Doppler ultrasound, duplex ultrasound, ankle-brachial pressure index), identifying 72 patients with purely arterial or purely venous ulcers of the lower extremities. We included only those with standardized photographic wound documentation in the study (see Table 2).

**Figure 1:**
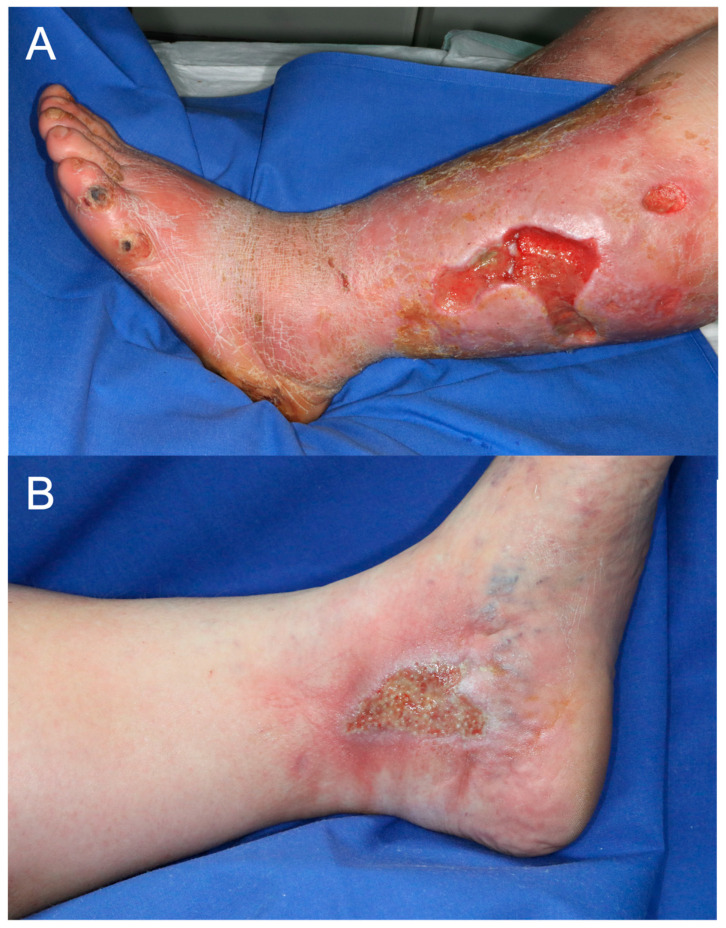
Example images of (**A**) an arterial ulcer and (**B**) a venous ulcer of the lower extremities.

The final dataset included 607 images in .jpg format (198 arterial and 409 venous ulcer images). The images were of varying sizes, with the average height and average width being 833 and 742 pixels, respectively. There was a repetition of patients with wound images taken at various stages of wound healing. However, no wound in its form was repeated twice. Images from the same patients were taken on different dates. Example images of unprocessed wound documentation for each ulcer class are shown in Figure 1. The images were randomly selected and represent the original format without any preprocessing.

### 2.2. Preprocessing

To enhance the focus on the region of interest (ROI), all images were manually cropped by delineating the wound and applying a 10% offset on all sides. This process effectively removed identifiable patient information while retaining a narrow margin of surrounding skin to preserve clinically relevant context. By eliminating unnecessary background information, we ensured that the analysis remained focused on the wound.

Given the color-dependent nature of wound images and their capture under controlled conditions, we employed a data augmentation process designed to enhance dataset variety without increasing its size, thereby supporting model robustness. We opted for weak augmentations to maintain the integrity of the visual data. Each image was resized to 224 × 224 pixels and normalized using a mean of 0.5 and a standard deviation of 0.5. During training, augmentations such as horizontal flip, vertical flip, and random cropping were applied with a 10% probability for each transformation. These augmentations were implemented dynamically at runtime, meaning they were randomly applied each time an image was accessed, modifying the sample without creating additional entries in the dataset. It is important to note that these augmentations, except for normalization and resizing, were not applied to the validation set or during inference.

A stratified train-test split was performed, with 10% of the dataset allocated to the test set to preserve class distribution. This split was held constant across all model training pipelines to ensure comparability of results.

### 2.3. Models

State-of-the-art deep learning models for image classification, namely ResNet50 [16], ResNeXt50 [18], ConvNeXt [19], EfficientNet B2 [20], and EfficientNet V2 [21], were selected for the task of ulcer classification, specifically to distinguish between venous and arterial ulcers. These models were chosen due to their proven performance in medical imaging and their architectural diversity, which allows for a comprehensive evaluation of classification performance across different CNN design principles. Each model was trained independently on the training dataset.

ResNet50 [20] is a deep convolutional neural network with 50 layers, introduced as part of the ResNet family, which uses residual learning to enable the effective training of very deep architectures. Its key innovation is the use of skip connections that allow gradients to flow more easily through the network, alleviating the vanishing gradient problem common in deep networks. This architecture enables ResNet50 to achieve high accuracy on image classification tasks while being more efficient and stable to train than traditional deep CNNs. ResNet50 has been widely used in applications ranging from transfer learning in general vision tasks [20] to specialized domains such as COVID-19 detection from chest X-rays [22] and skin cancer diagnosis from dermoscopy images [23].

ResNeXt50 [18] is a deep convolutional neural network that builds on the ResNet architecture by introducing the concept of cardinality, the number of parallel paths within a residual block, allowing it to capture more diverse feature representations without greatly increasing complexity. Instead of simply increasing depth or width, ResNeXt uses grouped convolutions to achieve a better trade-off between computational efficiency and model capacity. This design makes ResNeXt50 more accurate and flexible than standard ResNet50, particularly on large-scale image classification tasks like ImageNet. The model has been successfully applied in various classification problems, including skin lesion analysis and plant disease detection [24].

ConvNeXt [19] is a modern CNN architecture that updates the classic ResNet backbone using design strategies borrowed from Vision Transformers, such as large kernel sizes, LayerNorm, inverted bottlenecks, GELU activations, and the AdamW optimizer. These refinements help ConvNeXt achieve state-of-the-art performance on benchmarks like ImageNet while maintaining the efficiency and inductive bias of convolutional models. It has demonstrated its performance on a variety of tasks like detecting periapical lesions in radiographs [25], detecting vascular leukoencephalopathy from CT images [26], and domain-specific tasks such as rice grain type classification [27]. ConvNeXt variants. ConvNeXt is released in multiple capacity levels: Tiny (T), Small (S), Base (B), Large (L), and X-Large (XL). We use the Tiny (T) variant as our default backbone throughout this work.

EfficientNet-B2 [20] is a mid-sized member of the EfficientNet family introduced by Google AI, featuring compound scaling of network depth, width, and input resolution to optimize both accuracy and efficiency. The architecture uses MobileNetV2-style MBConv blocks [28] and was discovered via neural architecture search, making EfficientNet-B2 particularly efficient in parameter usage and FLOPs while delivering strong classification performance. EfficientNetB2 is known to outperform comparative models on various tasks like skin cancer classification and COVID-19 detection from ultrasound images [29].

EfficientNet-V2 [21] extends the original EfficientNet family by introducing several training- and architecture-level enhancements aimed at improving both convergence speed and predictive performance. It incorporates Fused-MBConv blocks, which combine depthwise and regular convolutions in early stages, and uses progressive learning—gradually increasing image resolution and regularization strength during training—to accelerate convergence and improve generalization. These innovations result in significantly faster training times (5×–11×) and higher accuracy, with EfficientNet-V2 models achieving up to 87.3% top-1 accuracy on ImageNet while maintaining parameter efficiency. EfficientNet-V2 variants have demonstrated superior performance across a range of classification tasks, including acute lymphoblastic leukemia detection [30], and liver fibrosis staging from MRI [31].

The architectures of the models were downloaded from Hugging Face in the configurations detailed in Table 3. To ensure a fair comparison and facilitate reproducibility, all deep-learning classifiers were trained using a unified protocol summarized in Table 4. A batch size of 32 and an initial learning rate of 1 × 10^−4^ were used, and models were trained for 50 epochs. Random weight initialization was carried out. We evaluated their performance using standard classification metrics, as described below. To explore model interpretability and better understand the decision-making process, we also applied Grad CAM to visualize the image regions most influential in each model’s predictions.

### 2.4. Performance Evaluation

In evaluating the performance of our Convolutional Neural Network (CNN) models for wound image classification, we employed several key metrics to ensure a comprehensive assessment. Accuracy was used as a primary indicator, measuring the proportion of correctly classified images out of the total number of images. This metric provides a straightforward measure of overall model performance. However, given the potential imbalance in wound image datasets, we also calculated Precision, which focuses on the correctness of positive predictions. Precision is particularly important in medical applications, as it reflects the model’s ability to minimize false positives, ensuring that when a wound is identified, it is indeed that wound type.

To further evaluate the model’s effectiveness, we incorporated the F1 Score, which balances Precision and Recall, the ability to find all relevant instances. The F1 Score is crucial in scenarios where both false positives and false negatives are costly, providing a single metric that captures the trade-off between these two aspects. To address class imbalance, we employed the ROC-AUC metric, which effectively evaluates the ability of the model to differentiate between classes by considering the trade-off between true positive and false positive rates. This metric is especially useful for imbalanced datasets, providing a comprehensive assessment of the model’s performance across all classes. Additionally, we utilized Gradient weighted Class Activation Mapping, Grad CAM, to enhance the explainability of our model’s decisions. Grad CAM generates visual explanations that highlight important regions in the image that contribute to the model’s prediction, offering insights into how the CNN interprets wound features. This approach not only aids in understanding model behavior but also builds trust in the model’s outputs, which is essential for clinical adoption.

## 3. Results

### 3.1. Performance Metrics

In our evaluation of various Convolutional Neural Network (CNN) architectures for wound image classification, we observed distinct performance metrics across different models. Results highlighted in Table 4 showcase the effectiveness of advanced CNN architectures in accurately classifying wound images. While there is room for improvement with the ConvNeXt model, the models from the EfficientNet family have exhibited superior performance even though they are relatively lightweight. The deeper models ResNet50 and ResNeXt50 also exhibited performance on par with EfficientNet family models.

Further analysis of precision and recall for all five models confirms strong performance across classes (Table 5)

### 3.2. Deep Learning Interpretability with Gradient-Weighted Class Activation Mapping (Grad-CAM), Precision Venous (%) and Precision Arterial (%) Refer to the Accuracy of the Model in Identifying Venous or Arterial Wounds Among All Its Predictions for Each Category

**Figure 2:**
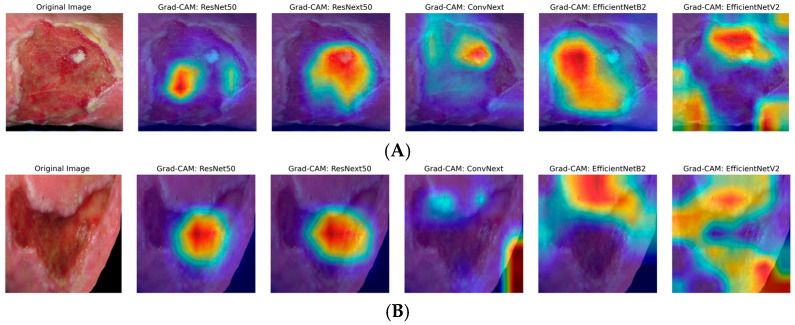
Grad-CAM visualizations for ulcer classification. (**A**) Venous ulcer; (**B**) Arterial ulcer: representative original images (far left) and Grad-CAM outputs for five different DL models: ResNet50, ResNeXt50, ConvNeXt, EfficientNetB2, EfficientNetV2 (from left to right). Red and yellow areas indicate regions highly relevant to the model decision, while blue and purple areas are less relevant.

The heatmaps generated by Grad-CAM are displayed in the figures below. The color ranges from blue to red and represents the significance value of the region, ranging from low (blue) to high (red), respectively, for predicting the etiology of the ulcer. Figure 2A illustrates the results for venous ulcers, and Figure 2B displays the model outputs for arterial ulcers.

As can be seen, only the models from the EfficientNet family have demonstrated dynamic focus, examining different parts of the images rather than fixating on the center. This dynamic attention suggests that these models are better equipped to adapt to variations in wound appearances, potentially contributing to their higher performance metrics. The insights gained from GradCAM underscore the importance of interpretability in medical image analysis, as understanding model behavior builds trust and aids in clinical decision-making.

## 4. Discussion

Our study demonstrates the significant potential of deep learning-based classification models for the differential diagnosis of arterial and venous ulcers. In particular, the EfficientNetV2 and ResNeXt50 CNN models exhibited a highly accurate differentiation between these two wound categories (97% and 98% accuracies). Huang et al. [33] presented a CNN model that classified five wound types (including arterial and venous ulcer wounds), achieving 96% and 86% accuracy in the venous and arterial ulcer classification tasks with an AUC (area under the curve) of 0.924 and 0.897, respectively. Although that impressive venous ulcer accuracy was based on only 26 venous wound images (with a total dataset of 2149 images), their five-class distinction was a much more complex task than our binary classification. Interestingly, their model outperformed the medical personnel significantly in all five wound categories [33]. Other binary classification studies [34,35] for distinct wound types or wound complications (e.g., maceration [35] using a CNN-based method similar to ours) can yield high accuracies if domain-appropriate data preprocessing is combined with modern CNN architectures. Patel et al. [36] used a multi-modal approach, demonstrating that including location data (exact lower leg region of the wound) can enhance performance in multi-class scenarios.

To make CNN-based models more transparent to explain AI and visualize relevant regions for CNN-based decision-making, we used Gradient-weighted Class Activation Mapping (Grad-CAM) [37]. Explainable AI (XAI) is undoubtedly a key aspect on the path to implementing AI in clinical routine, as it provides an intuitive insight into the decision-making process of neural networks. Visualization via Grad-CAM, in particular, makes it easier to understand why the AI assumes a venous or arterial etiology, for example. However, we have not conducted a direct comparison with experienced clinicians (e.g., dermatologists). In everyday practice, physicians do not make their decisions solely based on a “heat map.” They consider other clinical factors (e.g., Table 1) and assess the patient holistically. Pure image-based diagnosis with Grad-CAM analysis seems almost “limited” compared to this multifactorial clinical routine, but at the same time, it is even more impressive, as it achieves high levels of accuracy without additional information. Therefore, when faced with an image classification problem, a natural question arises about whether the model is genuinely identifying the etiology of the wound. A good visual explanation should be both class-discriminative (i.e., localizing the relevant regions necessary for a prediction) and high-resolution (i.e., finding the fine details in an image) [38]. Our analysis of the Grad-CAM results indicated that certain models, such as EfficientNetV2, dynamically concentrated on the ulcer borders and surrounding tissue, suggesting they incorporate contextual features like “punched-out” wound borders or skin discoloration in their decision-making. In contrast, other models, such as ResNeXt50, focused on the central wound bed, possibly highlighting ulcer depth and tissue composition. While Grad-CAM and LIME (Locally Interpretable Explanations and Model-Independent) [16] are qualitative heat-map approaches to highlight regions of the image that strongly influence the final prediction, Lo et al. found SHAP (SHapley Additive exPlanation) [17,39], the most effective and more detailed for providing quantitative per-pixel importance scores [14]. Therefore, the use of qualitative and quantitative XAI techniques to interpret deep learning models is a crucial part of validating and trusting these models in clinical practice [40].

Because our data was collected from a single-center cohort, future research should collect larger, multicenter datasets that include diverse imaging conditions (lighting, camera angle, background, distance) and patient demographics to improve generalizability and robustness. Additionally, we only considered the simplest scenario, binary classification of arterial versus venous ulcers. Real-world wound care involves distinguishing among mixed arterial–venous ulcers, diabetic foot ulcers, pressure ulcers, and other causes. Therefore, expanding and balancing the label set will be crucial. Furthermore, our analysis involved manually cropping images prior to processing, based on the assumption that images would primarily contain the wound and some immediate background. This approach is supported by existing literature and algorithms capable of automatic wound detection and cropping [15,41]. Additionally, we employed EfficientNet in its standard configuration to establish a robust and reproducible baseline for comparison, focusing on the effects of dataset quality, preprocessing, and augmentation without introducing architecture-specific confounders. Although attention modules like Squeeze-and-Excitation (SE) blocks [42] could improve feature weighting, they also add additional parameters and complexity, which may lead to overfitting—particularly with our small and unbalanced dataset. Finally, our models relied solely on wound photographs, excluding additional clinical data such as lesion localization, vascular imaging, patient characteristics, ankle–brachial index, or laboratory markers. Incorporating these data streams would provide richer context and likely enhance diagnostic accuracy. Addressing these limitations will help develop more accurate, robust, and clinically useful decision-support systems for ulcer assessment.

## 5. Conclusions

Chronic vascular ulcers impose a significant clinical and economic burden, and timely, precise differentiation among various etiologies is crucial for selecting effective treatment. This study aimed to determine whether modern convolutional neural networks can provide reliable, interpretable image-based support for this decision. We evaluated five CNNs (ResNet50, ResNeXt50, ConvNeXt, EfficientNetB2, and EfficientNetV2) for image-based differentiation of venous and arterial ulcers and used Grad-CAM to visualize the reasoning of the model. ResNeXt50 performed best with a macro-average accuracy of 97.85% and ROC-AUC of 0.9966, followed by EfficientNetV2 (96.77%) and ResNet50/EfficientNetB2 (95.70%). ConvNeXt (Tiny) lagged behind at 72.04%. Grad-CAM revealed model-specific attention patterns consistent with our metrics. The EfficientNet models displayed dynamic, context-aware focus, shifting between ulcer borders, perilesional skin changes, and the wound bed, while ResNext50 tended to fixate mainly on the center of the wound bed. This behavior aligns with the highest precision of EfficientNetV2 and supports the clinical plausibility of its decisions. These findings indicate that lightweight, explainable classifiers can support decision-making in the clinical setting. Limitations include a retrospective, single-center dataset of 607 images and a binary classification task. Future work will expand to multicenter and multiclass settings, incorporate segmentation, localization, and conduct prospective external validation.

## Figures and Tables

**Table 1:** Differential diagnosis between venous and arterial ulcers.

Characteristics	Venous Ulcers	Arterial Ulcers
Etiology	Sustained venous hypertension	Arterial insufficiency (ischemia)
Localization	Medial/lower leg	Toes, feet, lateral ankle, pressure points
Wound edge	Gently sloping	Punched-out, well-demarcated, deep
Wound bed	Red, granulating tissue with slough	Pale, dry, necrotic, exposed tendons
Exudate	Moderate-to-heavy	Minimal
Wound shape	Irregular	Regular, round
Pedal pulses	Present and normal	Diminished or absent
Surrounding skin	Edema, hemosiderin staining, lipodermatosclerosis	Shiny, cool, hair loss, pallor, cyanosis

**Table 2:** Inclusion and exclusion criteria.

Inclusion Criteria	Exclusion Criteria
Patients with a confirmed diagnosis of arterial or purely venous ulcers of the lower extremities	Mixed arterial–venous ulcers.
Availability of photographic wound documentation under standardized conditions	Ulcers of other etiologies.

**Table 3:** Selected Convolutional neural network architectures and weight references.

Model	Model Version	Hugging Face Configuration
ResNet50 [32]	Vanilla	resnet50.a1_in1k
ResNeXt50 [18]	Vanilla	resnext50_32x4d.a1h_in1k
ConvNeXt [19]	Tiny	convnextv2_tiny.fcmae_ft_in1k
EfficientNetB2 [20]	Vanilla	efficientnet_b2.ra_in1k
EfficientNetV2 [21]	Small	efficientnetv2_rw_s.ra2_in1

**Table 4:** Hyper-parameters for training classification models.

Hyper-Parameter	Value
Image Size	224 × 224
Batch Size	32
Learning Rate	1 × 10^−4^
Epochs	50

**Table 5:** Classification performance in %.

CNN Model	Macro-Avg Acc.	Precision (%)		Macro-Avg F1	ROC-AUC
		Venous	Arterial		
ResNet50	95.70	98.18	92.11	95.51	0.9883
ResNeXt50	97.85	98.25	97.22	97.73	0.9966
ConvNeXt (Tiny)	72.04	69.62	85.71	64.44	0.9381
EfficientNetB2	95.70	98.18	92.11	95.51	0.9744
EfficientNetV2	96.77	95.00	100.00	96.77	0.9810

## Data Availability

For data supporting reported results, please contact the corresponding author.

## Abbreviations

The following abbreviations are used in this manuscript:

AI	Artificial Intelligence
CNN	Convolutional neural networks
DCNN	Deep convolutional neural networks
Grad-CAM	Gradient-weighted Class Activation Mapping
PAD	Peripheral artery disease
CVI	Chronic venous insufficiency
